# Supplementary material for: Loss-of-Function Variants in SUPT5H as Modifying Factors in Beta-Thalassemia
Source: Int J Mol Sci. 2024 Aug 16;25(16):8928. doi: 10.3390/ijms25168928 (PMC11354595; doi:10.3390/ijms25168928)
Supplement: Supplementary file 1 [file ijms-25-08928-s001.zip › ijms-3114481-supplementary.pdf]

Table S1. *SUP5TH* MUTATIONS ASSOCIATED WITH INCREASED HEMOGLOBIN A$_2$ LEVEL

Individual	Gender	Age	Hb (g/L)	Hct (l/L)	MCV (fl)	MCH (pg)	RBCx 10^{12}	HBA$_2$% (CE)	Hb F(%)	*HBA* genotype	*HBB* genotype	*SUPT5H* mutation	Molecular effect	Reference
Dutchfamily1F1-II.1	M	66	111	0.4	91	25.3	4.39	5.1	*	normal	normal	c. [458+1G>A];[=]	Splice donor site	Ahlem Achour et al, 2020
DutchfamilyF1-II.2a	M	65	*	*	*	*	*	5.7	*	normal	normal	c. [458+1G>A];[=]		
DutchfamilyF1-II.3	F	62	108	0.32	84	28	3.83	5.7	*	normal	normal	c. [458+1G>A];[=]		
DutchfamilyF1-III.2	F	42	111	0.35	79	25.6	4.35	4.7	*	normal	normal	c. [458+1G>A];[=]		
DutchfamilyF1-III.3	F	40	116	0.33	79	25.4	4.23	4.8	*	normal	normal	c. [458+1G>A];[=]		
DutchfamilyF1-III.4	F	3	*	0.37	82	*	*	5.5	*	normal	normal	c. [458+1G>A];[=]		
DutchfamilyF1-IV.3	M	12	95	0.31	71	21.6	4.43	5.2	*	normal	normal	c. [458+1G>A];[=]		
DutchfamilyF1-IV.4	M	12	114	0.35	73	24.2	4.77	5.2	*	normal	normal	c. [458+1G>A];[=]		
DutchfamilyF1-IV.2	M	15	140	0.4	73	22.9	5.42	5.7	*	*HBA2*:c.[96-2A>G];[=]	normal	c. [458+1G>A];[=]		
DutchfamilyF1-III.1	M	54	142	0.46	81	24.8	5.72	2.4	*	*HBA2*:c.[96-2A>G];[=]	normal	normal		
DutchfamilyF1-IV.1	F	18	109	0.35	73	23.3	4.73	2.5	*	*HBA2*:c.[96-2A>G];[=]	normal	normal		
DutchfamilyF1-I.1	F	85	131	0.39	87	29	4.52	2.8	*	normal	normal	normal		
DutchfamilyF1-IV.5	M	6	127	0.37	80	26.2	4.62	2.5	*	normal	normal	normal		
Dutchfamily2F2-I.2	F	46	136	0.39	77	27.7	5.01	5	*	normal	normal	c.[2259-3C>A];[=]	Splice acceptor site	
DutchfamilyF2-II.3	F	16	135	0.41	77	25.8	5.31	4.7	*	normal	normal	c.[2259-3C>A];[=]		

Individual	Gender	Age	Hb (g/L)	Hct (l/L)	MCV (fl)	MCH (pg)	RBCx 10^{12}	HBA$_2$% (CE)	Hb F(%)	*HBA* genotype	*HBB* genotype	*SUPT5H* mutation	Molecular effect	Reference
DutchfamilyF2-II.4	M	11	134	0.39	72	24.2	5.39	4.9	*	normal	normal	c.[2259-3C>A];[=]	Splice acceptor site	
DutchfamilyF2-I.1	M	44	172	0.5	94	32.2	5.33	2.6	*	normal	normal	normal		
DucthfamilyF2-II.1	F	22	138	0.42	93	30.6	4.49	2.9	*	normal	normal	normal		
DutchfamilyF2-II.2	F	18	143	0.43	88	29	4.91	2.7	*	normal	normal	normal		
Wlt.1	M	65	121	0.35	77	26.2	4.59	5.3	*	normal	normal	c.[1979del;[=]		
Wlt.2	M	39	138	0.43	76	24.5	5.65	5.1	*	normal	normal	c.[1979del;[=]	p.(Gly660Valfs*6)	
Li	M	54	148	0.46	80	25.8	5.77	4.7	*	normal	normal	c.[1782_1785del];[=]	p.(Ile594Metfs*2)	
TW	M	6	113	0.38	79	23.7	4.75	5.2	*	normal	normal	c.[2725del];[=]	p.(Gln909Argfs*45	
S(French)	F	46	140	*	78	25.4	*	4.8	*	normal	normal	c.[817del];[=]		
Cl(French)	F	17	123	*	71	22.2	*	5.2	*	normal	normal	c.[817del];[=]	p.(Leu273*)	
GreekfamilyF3-I.1	F	*	97	0.32	57	17.1	*	8.5	*	normal	c. [118C>T];[=]	c. [1374+2T>C];[=]		
GreekfamilyF3-II.2	F	*	97	0.31	57	17.7	*	11.1	*	normal	c. [118C>T];[=]	c. [1374+2T>C];[=]		
GreekfamilyF3-II.3	M	*	149	0.44	75	25.3	*	5	*	normal	normal	c. [1374+2T>C];[=]		
GreekfamilyF3-III.1	M	*	121	0.36	70	23.1	*	5.3	*	normal	normal	c. [1374+2T>C];[=]	Splice donor site	Ahlem Achour et al, 2020
GreekfamilyF3-II.4	F	*	123	0.38	67	21.5	*	5.6		-α3.7/αα	c. [118C>T];[=]	normal		
GreekfamilyF3-I.2	M	*	139	0.41	90	30.1	*	1.7	*	normal	normal	normal		
GreekfamilyF4-III.1	M	2	102	0.31	46	15	*	12.4	*	normal	c.[92+1G>A];[=]	c.[1741_1744dup];[=]		
GreekfamilyF4-I.1	M	58	127	0.4	75	23.7	*	5.2	*	normal	normal	c.[1741_1744dup];[=]		
GreekfamilyF4-II.2	F	33	119	0.36	69	22.5	*	5.8	*	normal	normal	c.[1741_1744dup];[=]	p.(Arg582Glnfs*21)	
GreekfamilyF4-II.1	M	31	131	0.4	58	19.1	*	5.4	*	normal	c.[92+1G>A];[=]	normal		

Individual	Gender	Age	Hb (g/L)	Hct (l/L)	MCV (fl)	MCH (pg)	RBCx 10^{12}	HBA2% (CE)	Hb F(%)	HBA genotype	HBB genotype	SUPT5H mutation	Molecular effect	Reference
L1	*	*	117	*	80.8	25.5	4.59	5.5	1	normal	normal	del(chr19:39936531-40030719)		
L2	*	*	118	*	77.2	26.3	4.48	5.2	0.7	normal	normal	c.142G>T	(p.Glu48*)	
L3	*	*	108	*	72.8	23.9	4.52	5.9	0	-α3.7/αα	normal	c.631C>T	(p.Gln211*)	
L4	*	*	127	*	75.9	27.5	4.61	5.7	0.4	normal	normal	c.1405del	(p.Arg469Glufs*10)	Lou et al, 2023
L5	*	*	106	*	74.7	24.6	4.3	5.9	0	normal	normal	c.1855C>T	(p.Arg619*)	
L6	*	*	93	*	80.7	27.3	3.41	5.7	0	normal	normal	c.1901dup	(p.Met635Hisfs*19)	
L7Family1.1	*	*	*	*	*	*	*	*	*	normal	normal	c.2823_2850dup		
L7Family1.2	*	*	*	*	*	*	*	*	*	normal	normal	c.2823_2850dup		
L7Family1.3	*	*	*	*	*	*	*	*	*	normal	normal	c.2823_2850dup	(p.Ser951*)a	
X Family1.1	M	57	151	*	92.6	32.7	*	2.7	*	normal	normal	normal		Xiao et al,2023
X Family1.2	F	56	119	*	76.5	23.5	*	6.4	*	normal	normal	c.193C >T		
X Family1.3	F	33	120	*	82	26.4	*	5.8	*	normal	normal	c.193C >T	p.Arg65*	
C Family1.1	F	30	*	*	85.4	28.5	*	6.3	*	normal	normal	c.2368C>T		Charnay et al, 2022
C Family1.2	F	*	128	*	87.1	29.6	*	4.6	*	normal	normal	c.2368C>T	p.(Gln790*)	
G155739H (KR)	*	*	118	*	80.8	26	*	5.6	0.7	normal	normal	c.1855C>T	p.R619X	
G111157V	*	*	109	*	85	26.7	*	5.7	0.8	normal	normal	c.1789 A>T	p.L597X	
LM- 29525	*	*	127	*	80.5	25.6	*	5.6	0.5	normal	normal	c.1993_1994del	p.M665Efs19X	
29490	*	*	115	*	80.5	27.9	*	3.6	1.1	normal	normal	c.3034T>A	p.C1012S	
G109918F	*	*	91	*	81.2	27.1	*	4.8	0.4	normal	normal	c.2507 A>G	p.Y836C	Martell et al, 2023
G115621Q	*	*	142	*	79.4	28.1	*	4.4	0.6	normal	normal	c. 2245 C>T	p.R749W	
G130312A	*	*	120	*	86.2	28.4	*	4.6	0.3	normal	normal	c.delG2247	p.L750SfsTer11	
G1158492N	*	*	117	*	89.6	29.6	*	4.8	1.2	normal	normal		EXON 21 -2 A>G	
G199864F	*	*	142	*	74.4	23.5	*	5.4	0.4	normal	normal		p.E455Dfs*23	

*No information given
